# Khat use and intimate partner violence in a refugee population: a qualitative study in Dollo Ado, Ethiopia

**DOI:** 10.1186/s12889-020-08837-9

**Published:** 2020-05-12

**Authors:** Vandana Sharma, Stephanos Papaefstathiou, Samuel Tewolde, Adaugo Amobi, Negussie Deyessa, Bridget Relyea, Jennifer Scott

**Affiliations:** 1 Harvard T.H. Chan School of Public Health, 677 Huntington Avenue, Boston, MA 02115 USA; 2 École des Hautes Études en Santé Publique (EHESP), Paris, France; 3 Women and Health Alliance International Addis Ababa, Ethiopia and Paris, Paris, France; 4 Massachusetts General Hospital, Boston, MA USA; 5 Harvard Medical School, Boston, USA; 6 Addis Ababa University School of Public Health, Addis Ababa, Ethiopia; 7 Beth Israel Deaconess Medical Center, Boston, USA

**Keywords:** Intimate partner violence, Somalia, Substance use, Khat use, Qualitative data, Refugees, Humanitarian context

## Abstract

**Background:**

Intimate partner violence (IPV) is the most common form of gender-based violence affecting women and girls worldwide and is exacerbated in humanitarian crises. There is evidence that substance use is associated with male perpetration of IPV. Consumption of khat —a plant containing amphetamines traditionally chewed in the horn of Africa and legal in some countries including Ethiopia—may increase risk of IPV toward women. This analysis aimed to assess perceptions on khat use among Somali refugees in Dollo Ado, Ethiopia and its association with IPV to inform an IPV and HIV prevention intervention.

**Methods:**

A descriptive qualitative study comprising individual interviews (*n* = 30) and focus group discussions (*n* = 10) was conducted in Bokolmayo refugee camp in Dollo Ado, Ethiopia in October 2016. A purposive sample of male and female Somali refugees, religious and community leaders, and service providers (*n* = 110 individuals; 44 women and 66 men) was included. Trained interviewers from the camp conducted the interviews and discussion, which were audio recorded, transcribed and translated. A content analysis was conducted on coded excerpts from the transcripts to identify factors contributing to IPV toward women, including khat use.

**Results:**

Participants reported that displacement has resulted in limited employment opportunities for men and increased idle time, which has led to increased khat use among men as a coping mechanism. Male khat use was perceived to be associated with perpetration of physical and sexual IPV through several mechanisms including increased anger and aggression and enhanced sexual desire. Khat use also contributes to intra-marital conflict as money allocated for a household is spent on purchasing khat.

**Conclusion:**

Khat use should be addressed as part of IPV prevention programming in this context. Livelihood interventions and other strategies to improve economic conditions, should be explored in collaboration with refugee camp authorities and community leaders as a potential avenue to mitigate the impact of khat use on women and families.

## Background

Substance use and intimate partner violence (IPV) are important linked global health issues. Intimate partner violence, defined as “behavior by an intimate partner or ex-partner that causes physical, sexual or psychological harm, including physical aggression, sexual coercion, psychological abuse and controlling behaviors” [1], has adverse health, social and economic consequences for women and their families [2–8]. Globally, 30% of women have experienced lifetime physical and/or sexual IPV [1], and IPV prevalence is higher in humanitarian crises [9, 10].

Current research identifies substance use as being associated with IPV in several ways. Substance use is a risk factor for male perpetration of IPV [11–14], and women experiencing violence may use drugs or alcohol to cope with the trauma of an abusive relationship [15, 16]. Displacement may exacerbate substance use [17–19] but evidence on substance use among displaced populations is limited. Substance use has been described as a negative coping mechanism among refugee men living in camps, and an underlying risk factor for IPV perpetration [18]. There is a near absence of evidence on substance use interventions for displaced populations [17, 19], but substance use may be an important modifiable risk factor to target in IPV prevention interventions in humanitarian contexts.

There is even less evidence on use of locally available substances including khat (*Chata Edulis*), which has been understudied and largely ignored in the literature [20]. Khat is a plant native to East Africa and the Arabic peninsula with known stimulant properties [21, 22]. Khat is chewed widely in East Africa where its use is legal, and regarded as a symbol of elevated social status [23–25]^.^ The current estimate of khat use in the region is over 20 million daily users [26] but global use may be even higher. While research on khat use is limited, some evidence suggests adverse health impacts including behavioral and mental health problems [26–28]. Several studies have also demonstrated associations between khat use and IPV perpetration [29, 30], including among Somali populations [31]. However, there are limited data on mechanisms through which khat use may lead to IPV, and how this relationship may be influenced by displacement. The aim of this analysis was to assess perspectives on khat use and describe the pathways through which khat use may be associated with IPV among Somali refugees in Dollo Ado, Ethiopia to inform the development of an IPV and HIV prevention intervention.

## Methods

A descriptive qualitative study was conducted in 2016 in Bokolmayo refugee camp to explore factors that contribute to IPV in this setting. Bokolmayo is one of five refugee camps near Dollo Ado, Ethiopia, a small town bordering Somalia. The camp, opened in 2010, and at the time of the study, over 200,000 Somali refugees were registered in the five camps, with over 40,000 refugees residing in Bokolmayo [32].

The study was designed to inform the adaptation of an IPV and HIV prevention intervention, Unite for a Better Life, to a humanitarian context and to develop intervention content tailored for displaced populations. The intervention and its theory of change were designed around an ecological model of violence that recognizes that multiple factors operate at various levels (individual, relationship, community, and society) to influence IPV risk [33]. The intervention comprises a multi-session curriculum which includes facilitated discussions, activities and exercises on such topics as gender norms, sexuality, power in relationships, violence and conflict resolution, and HIV transmission. The content is delivered by trained facilitators to groups of women, men and couples in participatory group sessions [34] or through podcast episodes (MP3 audio files) [35].

In 2016–2018, the curriculum was adapted for use among a Somali refugee population living in refugee camps in Dollo Ado, Ethiopia. This process comprised consultations with women and men refugee community members and other key stakeholders (2014), assembly of a study community advisory board and formative qualitative research (2016), adaptation of the intervention theory of change and creation of new curricular content (2017), and piloting of the intervention among a random sample of Somali women and men refugees in Bokolmayo refugee camp (2018). Concurrently, program content was adapted to create podcast episodes and piloted in listening centers in the refugee camp (2017–2019). The quantitative baseline and endline pilot data will be presented elsewhere, but indicate high prevalence of past-year IPV experience among women and IPV perpetration among men. The quantitative baseline data also indicate that khat use among Somali men refugees in the study site was common with 40% of men reporting current khat use.

The qualitative study included a purposive sample comprising women and men refugee community members, community elders, religious leaders, service providers and organization staff, as well as host community members (individuals living in Dollo Ado who have not been displaced). Participants were recruited in consultation with organizational partners, the United Nations High Commissioner for Refugees (UNHCR), the Administration for Refugee and Returnee Affairs (ARRA), and the study’s community advisory board.

Data collection methods included in-depth interviews (IDIs), focus group discussions (FGDs), and group-based participatory learning activities (PLAs) (See Table 1). For this paper, we present data from the IDIs and FGDs only.

**Table 1:** Summary of types and numbers of qualitative data collection methods

**Methodology**	**# of interviews / discussions**	**# of participants**
In-depth Interviews	30	30
Focus Group Discussions	10	80
Participatory Learning Activity* (Free Listing and Vignettes)	10	81
Participatory Learning Activity* (Community Mapping)	3	24
TOTAL	**53**	**215**

*data were excluded from this analysis

Study participants included women and men aged 15 and above, who identified as Somali refugees residing in Bokolmayo for at least 6 months. The age inclusion criterion took into account several determinants. First, child marriage (marriage before the age of 18) is a common practice in Somali culture [36], and second, the WHO multi-country study on women’s health and domestic violence conducted in Ethiopia reported higher levels of violence within the 15–19 age group compared to other ages [37]. The inclusion criteria for organization staff and/or service providers included: adult (aged 18 and older) female and male staff from community-based organizations (CBOs) operating in Dollo Ado, who have worked for at least 1 yr with the organization. The inclusion criteria for community leaders and elders included: adult (aged 18 and older) female and male community leaders and/or religious leaders in Dollo Ado, who have been in Bokolmayo camp for at least 6 months. The exclusion criterion for all three study groups was any illness or condition that limited the participant’s capacity to communicate. Khat use was not an inclusion criterion for participation in the qualitative study given its broader aims to inform the adaptation of the entire intervention.

### Ethical considerations

Verbal informed consent was obtained from all participants. During the consent process, the study participants were informed of their right to decline participation, to terminate participation at any time, and that refusal or termination would not impact their eligibility to receive services in the camp. For participants under the age of 18, parental consent was not obtained to ensure privacy. Parents may not be present to give their consent, as families may be separated during displacement. Furthermore, a request for parental consent may place participants at risk if the sensitive nature of the study topic is revealed. This study was approved by institutional review boards at Beth Israel Deaconess Medical Center (BIDMC) in Boston, USA, as well as the Addis Ababa University (AAU) in Ethiopia. Permissions to conduct the research were also obtained from UNHCR, ARRA, and the local community advisory board comprised of community leaders and stakeholders, who reviewed the study protocol and provided input on study procedures and tools. The onsite research supervisors, interviewers and facilitators signed a code of conduct to ensure protection of study participants, integrity of data collection and fidelity to the study protocol. For FGD participants, facilitators emphasized that participants must also respect the confidentiality of the other participants and not disclose information from the discussion outside of the group. All participants were assigned a unique identifier to protect anonymity.

### Data collection

Semi-structured questions were developed for the IDIs and FGDs to assess factors that contribute to IPV, including gender, social, religious and cultural norms and the influence of displacement on behaviors and IPV. Questions related to khat focused on perceptions of ways in which khat use may be associated with IPV in this context. Examples of khat-related questions include: 1) Is chewing khat a common practice in your community? Was it practiced when you were in Somalia?, 2) How does a person react before and after chewing khat?, 3) What are some of the effects of chewing khat? (Probe about money problems, health problems, conflict with family, friends and partner, problems with the law).

Three female and seven male interviewers fluent in Somali and local dialects were recruited from the refugee camp and completed a six-day training. The training focused on qualitative methods, interviewing techniques, and protection of human subjects along with risk mitigation. Facilitators and interviewers operated in pairs for security reasons. One facilitated the discussion or conducted the interview, while the other operated as a note-taker. Data collection was conducted in private settings and audio-recorded. Supervisors provided support and quality assurance. Interviewers completed a debriefing template following each interview including notes about the data collection process. Interviews were between 40 and 90 min, while FGDs were between 120 and 220 min. A list of local medical, legal and other relevant support services was given to participants upon completion and referrals for psychological support were also provided.

### Data analysis

Audio recordings of the IDIs and FGDs were transcribed verbatim in the original dialect and translated to English. The transcripts were uploaded into the Dedoose qualitative analysis software system (version 7.6.6). A coding framework was developed a priori by the study team (VS, JS, ST) for the main analysis and followed a deductive approach based on the structure of the IDI and FGD guides. The coding framework included seven broad areas including: 1) Safety and security in the camp, 2) Cultural practices and ceremonies, 3) Tradition, community and gender norms, 4) Intimate partner violence against women, 5) Non-partner violence against women, 6) Violence against men and boys, and 7) Violence response. Each of these broad codes also included a series of subcodes. Khat was a subcode within this framework.

For the current analysis, conventional content analysis [38] was conducted on coded excerpts from the transcripts to identify pathways through which khat use may be associated with to IPV toward women. FGD and IDI transcripts were first reviewed and coded using the coding framework (VS, SP, AA, JS). The PLA transcripts were reviewed, but there were minimal data on khat and thus were not included in this analysis. Coded excerpts specific to khat were extracted and categorized according to emerging themes and subthemes and were analyzed. This thematic analysis identified five prominent themes related to khat consumption and its relationship with IPV as well as how this relationship may have been changed by displacement in this population.

## Results

In total, 30 IDIs and 10 FGDs were conducted (*n* = 110 individuals; 44 women and 66 men).

### Participant characteristics

In total, 30 participants (13 women, 17 men) were individually interviewed and 80 (35 women and 45 men) participated in FGDs (See Table 2). Participants in the IDIs ranged from ages 17 to 70 years, and their average length of time in the camp was 7.3 years (6.8 years for women, and 7.8 years for men). Most respondents were married, and all identified as Muslim. The average number of years of formal education was 5.6 years (4.3 years in women, and 6.6 years in men), but these data were skewed by education levels of the health workers and NGO staff in the sample. The demographic characteristics were similar in the FGD participants (See Table 3).

**Table 2:** Demographic data for in-depth interviews

**Participant Demographics**	**Refugee Community Members**	**Elders / Religious Leaders**	**Health Workers**	U**N / Non-Governmental Organization**s	**Community- Based Organization**s	**Policy-makers**	**Host Community Members**	**Total**
	**N (%)**	**N (%)**	**N (%)**	**N (%)**	**N (%)**	**N (%)**	**N (%)**	**N (%)**
# of interviews	16	4	2	2	2	2	2	30
Nationality								
Somali	16 (100)	4 (100)	2 (100)	0 (0)	2 (100)	0 (0)	0 (0)	24 (80
Ethiopian	0 (0)	0 (0)	0 (0)	2 (100)	0 (0)	2 (100)	2 (100)	6 (20)
Sex								
Female	8 (50)	0 (0)	0 (0)	2 (100)	1 (50)	1 (50)	1 (50)	13 (43)
Male	8 (50)	4 (100)	2 (100)	0 (0)	1 (50)	1 (50)	1 (50)	17 (57)
Age (mean, range, in years)	31.7 (17–62)	61.3 (51–70)	45.5 (45–46)	22.5 (20–25)	36.5 (32–41)	31 (19–43)	40.5 (37–44)	36.8 (17–70)
Marital Status								
Single	5 (31)	0 (0)	0 (0)	2 (100)	0 (0)	0 (0)	0 (0)	7 (23)
Married	11 (69)	4 (100)	2 (100)	0 (0)	2 (100)	1 (50)	2 (100)	22 (73)
Separated	0 (0)	0 (0)	0 (0)	0 (0)	0 (0)	1 (50)	0 (0)	1 (3)
Length of time in camp (Mean, range, in years)	6.8 (0.3–9)	7.6 (7–8)	7.5 (7–8)	1.7 (1–2.5)	8 (8)	8 (8)	N/A	7.3 (0.3–9)
Years of Education (mean, range, in years)	3.6 (0–15)	2 (0–8)	15 (14–16)	15 (15)	10 (8–12)	7.5 (7–8)	2.5 (0–5)	5.6 (0–16)

**Table 3:** Demographic data for focus group discussions

**FGD** **#**	**Participant Type**	**Total # of Women**	**Total #** **of Men**	**Country of Origin**	**Age Range** **(years)**	**Length of Time in Camp** **(years)**
1	Male (aged 15–25 years)	0	8	Somalia	17–25	1–8
2	Male & Female (26–45 years)	4	4	Somalia	27–45	6
3	Male (> 45 years)	0	8	Somalia	48–82	6
4	Female (15–25 years)	8	0	Somalia	17–25	7–8
5	Female (26–45 years)	8	0	Somalia	25–45	6–8
6	Female (> 45 years)	8	0	Somalia	45–49	5–8
Other Groups						
7	Clan / Religious Leaders / Elders	0	8	Somalia	25–80	6–8
8	Health Workers	1	7	Ethiopia	25–29	1–5
9	UN / Non-Governmental Organizations	2	6	Somalia & Ethiopia	21–49	1–6
10	Community-based Organizations	4	4	Somalia	27–50	8
Total		**35**	**45**			

Several key thematic areas emerged from the data.

#### Patterns of khat use and the impact of displacement

Several participants described khat use as common in Somalia prior to displacement. Numerous participants reported that men predominantly chew khat, while several referred specifically to women constituting a minority of khat chewers (answers ranged from “some” to percentage estimates between 0.1 to 10% of all khat chewers in Somalia). For example, one participant noted: “It is not too many … one [woman chewer] you may get, but not many.” (IDI 4, male refugee, > 45 years). On the other hand, one female refugee reported that she had never witnessed a woman chewing khat.

Several changes in khat use were reported as a result of displacement. First, several participants noted an overall increase in khat use in the refugee camp compared to pre-displacement. “Yes it was eaten previously and now it is eaten more here [in the camp]” (IDI 12, male refugee, 15–25 years). Several indicated that the prevalence has increased as men use khat as a coping mechanism. For example, one refugee stated that in the camp men chew khat because: “they have nothing in their hands … some do it because of their unemployment status.” (IDI 18, male refugee, > 45 years). Another respondent emphasized that men in the camp use khat as a means to deal with their stress: “They do believe [khat] to prevent and reduce stress.” (IDI 24, female, CBO, 26–45 years). Several participants described that displacement has led to reduced financial opportunities for men, while women are able to find paid informal work more easily, contributing to shifts in roles as women become financial supporters of the household. This was noted to contribute to an increase in khat use by men who face the stress of loss of livelihoods together with the renegotiation of gender roles within the household.

A few interviewees mentioned the post-displacement inclusion of women in khat chewing, and ascribed this to greater liberties in the camp. At the same time, several others described khat chewing by women as taboo or shameful in the camp: “Most of the time women are ashamed to chew khat, she is a mother of children.” (IDI 16 male refugee, 15–25 years). A different respondent (IDI 21, male health worker, > 45 years) suggested that a larger number of women use khat than generally thought, but that they do so in a non-visible manner through the addition of khat to tobacco used in shisha smoking. The respondent alluded to khat use being a new custom for women.

Several participants provided insights on the general mode of khat consumption pre- and post-displacement, illustrating the associated traditional rituals. Khat was reported to be traditionally chewed in teashops and cafeterias that allow people to gather for social exchanges, with the accompaniment of alcoholic and non-alcoholic beverages. One respondent noted that only men have the freedom to chew khat where they please, whether it be in the street or a teashop. Two participants noted that both genders gather in khat chewing places, while a different participant suggested women do not consume khat in mixed-gender gatherings but rather serve the coffee or tea.

#### Perceived behavioral changes associated with khat use

There was a common perception that khat use impacts an individual’s physical and mental health and behavior, and that there are differential effects when a user is under the influence versus experiencing withdrawal symptoms or effects from long-term use of khat.

Several respondents including refugee community members, health workers, CBO workers and clan leaders discussed perceived health impacts of khat use, including digestive problems such as constipation, headaches, destruction of teeth, and heart problems, while others were less specific but reported that it is detrimental to the health and body. For example, one participant explained: “[Khat] destroys the teeth, it also destroys to the other parts of the body, even if it is not visible … I have seen a man who has undergone an operation of the intestine as khat had accumulated in it” (IDI 27, male policy maker, 26–45 years). One religious leader stated: “The person is a human being and is not intended to chew [khat] leaves. Though people are saying it is a vegetable. But when you tear it has strong branches or has a hard stem and if that accumulate the stomach of the person, I think strongly that it harms his health (IDI 14, male religious leader, >45 years). One participant described a general loss of appetite due to khat chewing, while a different participant explained that a khat user “eats everything.” Several respondents referred to the disruptive impact of khat on sleep indicating that a chewer may “sleep for 24 hours,” or that it may be hard to wake one up after a khat-chewing session. One respondent described khat-related insomnia.

All participants who discussed khat use referred to behavioral changes when chewing khat, including behavioral changes brought on by extensive or long-term khat use. This was usually mentioned by comparing the user’s state of mind before and after khat chewing, with before described as “normal”, “good”, or “stable”, and after as prone to uncertainty, “wrong”, and unable to “coincide with family culture.” Numerous participants mentioned khat’s stimulant characteristics resulting in a hyperactive state; however, certain individuals reportedly become quieter under the influence of khat. It was also noted that khat users may find it intolerable to be proximal to family conversations, or unable to contribute to society. For example, one participant stated: “Majority of them [men in the camp] are useless and chew khat and share useless information.” (FGD 6, female refugees, > 45 years).

Several participants described a person intoxicated with khat as being in a state of madness, mindlessness, and prone to hallucinating. For example, one respondent stated: “The face changes and the mind changes.” (IDI 4, male refugee, > 45 years). A different respondent stated: “His mind changes, you don’t know whether he is mad or sane.” (IDI 10, female refugee, 15–25 years).

Many participants stated that khat chewing renders a person angry, while several participants said that this anger may manifest as physical aggression. According to a male clan leader this may coincide with a perceived enhancement of physical strength in a person high on khat: “he seems he is the strongest and becomes aggressive because his mind has changed, once he chews, he feels stronger and his mind changes.” (IDI 20, male clan leader, > 45 years). Several participants mention a change in morality upon the attainment of a khat high, as individuals under the influence are perceived to be more prone to “lying”, “making false promises”, and “stealing.”

Two participants indicated that upon withdrawal of khat, a khat user may exhibit anger. For example: “When he misses the khat he gets angry, always gets angry. If he does not chew, his mind will be out or absent” (IDI 4, male refugee, > 45 years).

#### Economic impacts of khat use on families and households

The majority of the responses referred to the financial implications of khat use in the camps both for the person using and for the family. Khat use was bidirectionally linked with unemployment. Participants described men using khat because they were unemployed and had too much idle time, while others described unemployment as a consequence of khat use. For example, several participants categorized men into those who work, and those who chew khat. One participant made a further categorization of men who are unable to work due to a disability, and those who are physically able yet spend their time chewing khat: “Men who are staying in Bokolmayo, they are those types, one he can’t work, he is handicapped, and one he doesn’t work and looks only for khat.” (FDG 5, female refugees, 26–45 years). Other participants expressed that chewing khat does not allow one to maintain employment. For example, one respondent stated: “[khat users] get angry, they become lazy, they don’t want to work.” (IDI 12, male refugee, 15–25 years).

The majority of participants noted that khat becomes a financial priority for the user, and that money allocated for the family’s survival goes to the procurement and consumption of khat. For example, one respondent explained: “If the person initiates chewing khat, he has zero money, all is used for khat. Then it might be that children go to bed hungry.” (IDI 3, female refugee, > 45 years).

One participant stated that a khat user is less likely to assume their duties in terms of providing for their family or spending time with their children: “ … as the behavior changes, the person’s income doesn’t increase, every time he is searching for khat if he doesn’t have it, it may happen to commit very bad things, lie, even some start theft. They become indebted with more money for buying the khat from sellers, or borrow from them. Then they may [cause] conflict and fight and it can affect the security of their family. Khat has an effect to [cause] conflict every time whether his parents, brothers or sisters, his children…He spends his time outside instead of with his children.” (IDI 23, female, Ethiopian organizational worker, 15–25 years). Two participants mentioned that a khat user may resort to begging to sustain the habit. “He becomes a thief and ever if he misses khat, he begs” (IDI 7, female refugee, 26–45 years).

Several participants described the financial costs of sustaining a khat habit. For example, one participant a female refugee, 26–45 years old, stated that the employment rate in the Bokolmayo camp is 20%, and that the maximum daily salary that can be achieved is 700 birr (~ 24 USD), while comparing this to the amount of 150 birr (~ 5 USD) needed each day to buy khat. Another participant, a Somali male health worker, estimates an individual’s earnings at 600 birr (~ 20 USD). From this, 300 birr (~ 10 USD) may be allocated to khat (an amount also noted by another respondent), plus an extra 100 birr to chew khat at a tea shop for beverages or the cover charge, leaving the family with the meager amount of 200 birr. Another male refugee 26–45 years old, explained that a quarter of a bundle of khat costs 75 birr (~ 3 USD), which is on average equivalent to the daily expenses for a family.

Several participants also mentioned that distributed food rations or meal cards may be monetized to procure khat. For example: “Some of them use their meal cards in exchange for khat and leave women to suffer in hunger … Their husbands chew khat and they buy khat using the meal card and conflicts arise. They [the wives] take jerrycans of water and beg others ‘please give me 10 Birr to feed my child’ while the man stays in the [khat] chewing places.” (IDI 22, male health worker, 26–45 years).

Debt was also described as being commonly incurred by khat users upon borrowing money from friends, family members, or when consuming khat on a credit-basis in tea shops. One male refugee, 26–45 years old, stated that the entire clan may be held accountable for one individual’s debt related to khat use. Others described a khat user’s debt affecting the credit of his wife who may then be unable to obtain necessary supplies from shops.

#### Associations between khat use and intimate partner violence

Numerous respondents described anger and aggressive behavior, and several specifically described physical violence towards wives as being related to khat use. Several noted that under the influence of khat, a man may beat his wife despite her adherence to traditional roles and norms. Several participants described specific circumstances that may elicit physical IPV. One respondent linked physical IPV to khat withdrawal symptoms: “Some men they may fight with their children and the wife when they don’t have khat, and when they have khat are happy.” (IDI 13, female refugee, 26–45 years). The same respondent explained the outcome if a wife asked her husband to stop chewing khat: “If she said that, he would fight and beat.” (IDI 13, female refugee, 26–45 years). Another respondent stated: “… as the man chews the khat he hallucinates … once he reaches the woman, if she returns any word, he beats.” (IDI 15, male refugee, 26–45 years). One respondent described women buying khat for their husbands in order to prevent IPV: “She buys him khat, and if he is not bought khat, there will be no peace.” (FGD 10, CBOs, 27–50 years).

Several participants stated that the financial struggles due to khat use along with absence from the family causes the responsibility of family support to shift to the spouse not consuming khat, and gives rise to intra-marital conflict and physical violence towards the wife. For example, “If he is drug addicted and his family hungry then he purchases khat instead of giving to his family and his wife asks income to feed children, then it’s violence” (FGD 2, mixed sex group, 26–45 years). Some participants suggested that women have become the economic backbone of their families, and by extension of the refugee community, due to being forced to seek employment at greater distances and to cater alone for their families’ needs because of their spouses’ khat use. Respondents described this as leading to a male khat user’s “re-negotiation” of the breadwinner role, and this causing intra-marital conflict. “It brings many problems in the relationship, because the manhood of the man is weakened on the side of the economy … The income which he may get, meant for the children or the family, is lost, then conflict and disagreement will come …” (IDI 25, female refugee, CBO, 26–45 years).

Several respondents described sexual IPV as a possible consequence of khat use. This was described as due to excessive sexual desire on the part of khat users. “When he chews khat, he wants to have sex every night and she cannot tolerate it … there are two issues, it is economic destruction of the family and desire for excessive sex and that are the biggest problems [with khat use].” (IDI 21, male health worker, > 45 years). The same participant, a male health worker, also suggested that women’s consumption of khat causes destruction of the family due to khat-driven infidelity, explaining that females are particularly susceptible to enhanced sexual desire when under the influence of khat. Another respondent described khat users returning home in the middle of the night after chewing all day and demanding sex, and referred to this as violence.

#### Social norms and perceptions on khat use and its regulation in the camp

In general, khat use was perceived as “not normal”, and khat users as “useless” individuals unable to contribute to the community and with whom a friendship cannot be maintained, and who “have given up on their livelihood”. One respondent stated that: “It is a problem to the community, it is in the bad culture.” (IDI 4, male refugee, > 45 years). Khat users were deemed to be a source of insecurity in the community, and several participants mentioned that khat use is forbidden by Islamic religion. For example, one participant stated: “First, khat is a drug, it is forbidden. Usually if the man has a wife and children, our religion doesn’t allow the drugs whether khat or cigarettes or bad behavior.” (IDI 8, male clan leader, > 45 years).

In terms of the regulation of khat use in the camps, several participants outlined that agents of law enforcement consume khat, and that corruption (often stemming from khat trade and use) among law enforcement impedes efforts to curb negative behavior related to khat. One respondent noted: “The law? Those in the law enforcement also chew.” (IDI 20, male clan leader, > 45 years).

## Discussion

Findings from this qualitative study suggest changes in patterns of khat use among Somali refugees in Dollo Ado, Ethiopia as a result of displacement. Khat consumption among men was perceived to have increased due to displacement and khat is used as a negative coping strategy to relieve stress associated with displacement, loss of livelihoods and camp conditions. Many men reportedly cope poorly with the continued poverty and high unemployment in this setting. As they struggle to provide for their families, they self-medicate with khat. These findings are broadly consistent with the limited existing evidence which suggests substance use increases in humanitarian emergencies [17–19, 39]. While an increase in khat use by women was also described in Dollo Ado, this was attributed to greater liberties in the camp rather than a means to cope with stress or trauma. However, further research among women khat chewers in this setting is needed to confirm this finding. The reported behavioral changes due to khat use, including withdrawal symptoms, as well as heightened anger, irritability and aggression and associated mental health issues also align with previous studies [26–28, 40] and underscore the need for public health interventions to address this problem.

The economic impacts of khat consumption in a context where families already struggle to make ends meet are immense and place already vulnerable families at greater risk and threaten their survival. The study finds that the links between khat use and economics are complex and bidirectional. While the economic situation in the camp exacerbates khat use, khat use drains family resources leading to greater poverty. One other study noted similar financial consequences of khat use in a refugee setting [18], but this area has not been well studied.

Importantly, our findings highlight the association between khat use and IPV and shed light on the mechanisms through which khat use may be a risk factor for IPV. Our findings are consistent with several other studies that report a significant association between male perpetration of IPV and substance use (including khat) [29–31, 39]. For example, one study among Somali refugee community workers in Dadaab refugee camp in Kenya reported khat use as a driver of GBV [39]. Another study in three refugee camps (South Sudan, Kenya, and Iraq) described substance use as a driver of IPV across all camps, with khat use linked to IPV specifically in Dadaab camp [18]. These studies however, do not attempt to assess associations between khat use and specific forms of IPV (physical versus sexual), and only one study reported on potential pathways through which khat use may elevate IPV risk [18]. Our findings therefore provide new insights into how khat use may increase risk of IPV and underscore a link between khat and both physical and sexual IPV.

The findings highlight three main channels through which khat use reportedly operates as a driver of IPV. First, physical IPV occurs directly through increased, anger and aggression brought on by khat use (often during periods of withdrawal or excessive use). A second direct pathway through which khat use leads to IPV is through elevated and excessive sexual desire which when coupled with aggressive behavior leads to sexual IPV. This pathway may also lead to physical violence if a woman refuses sexual advances. Finally, IPV may also occur more indirectly through a multi-step pathway related to the financial and economic consequences of khat use that threaten family survival. In this case, as household income, and sometimes food rations and other household resources, is spent on khat, intra-marital tension and conflict occur. Similarly, shifts in gender roles as women become the breadwinners while men spend time consuming substances rather than working or engaging with the family contribute to family tensions and violence. One other study also reported evidence supporting the third, indirect channel operating via the economic burden of khat use [18]. These findings highlight the importance of addressing substance use and misuse as part of IPV prevention programing; as such additional content on khat use, including harm reduction, was developed for the Unite for a Better Life in-person and podcast programs [34, 35].

The study has several strengths. First, the sample is diverse and includes women and men, religious leaders and service providers to capture differing perspectives. The inclusion of individual interviews and focus group discussions enabled assessment of majority views and group norms, as well as dissenting perspectives. Limitations include the fact that causal relationships could not be assessed using the qualitative methodology, and that comparative data were not collected from non-displaced populations in Somalia. Social desirability bias could have influenced responses, but interviewers were recruited from within the camp and were trained in strategies to build trust and rapport with respondents. External validity of the findings may be limited given the non-probability sampling approach. Sex and gender are important within this cultural context and were considered at all levels of the design and implementation of the study. A community advisory board provided guidance to ensure that gender and cultural factors were appropriately considered in this study. For example, we aimed to include an equal number of male and female participants in the data collection. However, the final totals include more men than women because some of the specific targeted groups (i.e. religious leaders, clan leaders) comprise mainly men in this context. Male and female data collectors were employed, but it was a challenge to recruit sufficient numbers of women for this position. Perspectives from both women and men were included in all thematic areas, but there was differential reporting based on gender, resulting from gendered patterns of khat use.

## Conclusion

In summary, this study provides evidence of the perceived negative consequences of khat use within this refugee context, including its link with both physical and sexual IPV through multiple pathways. The findings point to specific avenues for future programming and research. First, dedicated programming to address substance dependence in this setting should be prioritized. Given the limited evidence base on effective substance use programs for humanitarian settings, further research is need to develop and test such interventions. Programming to prevent IPV should address substance use and social support components or these elements should be implemented alongside broader prevention efforts to increase their effectiveness. Livelihood interventions and improved economic conditions may also be an important route to address khat use.

## Data Availability

The datasets generated and analyzed during this study are not publicly available since participants did not give consent for the public sharing of their information. However, summaries of the information are available from the corresponding author upon reasonable request. The interview guides are also available upon request.

## Abbreviations

AAU: Addis Ababa University
ARRA: Administration for Refugee and Returnee Affairs
BIDMC: Beth Israel Deaconess Medical Center
EHESP: École des Hautes Études en Santé Publique
FGD: Focus Group Discussion
HIF: Humanitarian Innovation Fund
IDI: In-depth Interview
IPV: Intimate Partner Violence
PLA: Participatory Learning Activity
UNHCR: United Nations High Commissioner for Refugees
WAHA: Women and Health Alliance International
